# The GAL/GALR2 axis promotes the perineural invasion of salivary adenoid cystic carcinoma via epithelial‐to‐mesenchymal transition

**DOI:** 10.1002/cam4.5181

**Published:** 2022-08-29

**Authors:** Jun Wang, Zihui Yang, Yuanyang Liu, Huan Li, Xiangming Yang, Wanpeng Gao, Qi Zhao, Xinjie Yang, Jianhua Wei

**Affiliations:** ^1^ State Key Laboratory of Military Stomatology and National Clinical Research Center for Oral Diseases, and Shaanxi Clinical Research Center for Oral Diseases, Department of Oral and Maxillofacial Surgery, School of Stomatology Fourth Military Medical University Xi'an China; ^2^ Senior Department of Neurosurgery First Medical Center, Chinese PLA General Hospital Beijing China

**Keywords:** epithelial‐to‐mesenchymal transition, GAL, GALR2, perineural invasion, salivary adenoid cystic carcinoma

## Abstract

**Background:**

Perineural invasion (PNI) is a typical pathological characteristic of salivary adenoid cystic carcinoma (SACC) and other neurotrophic cancers. The mechanism of the neural microenvironment controlling tumor progression during the PNI process is unclear. In the present study, we investigated the role and molecular mechanisms of nerve‐derived neuropeptide galanin (GAL) and its receptor (GALR2) in the regulation of PNI in SACC.

**Methods:**

Immunohistochemistry staining and clinical association studies were performed to analyze the expression of GAL and GALR2 in SACC tissues and their clinical value. Dorsal root ganglion or SH‐SY5Y cells were co‐cultured with SACC cells in vitro to simulate the interactions between the neural microenvironment and tumor cells, and a series of assays including transcriptome sequencing, Western blot, and Transwell were performed to investigate the role and molecular mechanism of GAL and GALR2 in the regulation of SACC cells. Moreover, both the in vitro and in vivo PNI models were established to assess the potential PNI‐specific therapeutic effects by blocking the GAL/GALR2 axis.

**Results:**

GAL and GALR2 were highly expressed in SACC tissues, and were associated with PNI and poor prognosis in SACC patients (p < 0.05). Nerve‐derived GAL activated GALR2 expression in SACC cells and induced epithelial‐to‐mesenchymal transition (EMT) in SACC cells. Adding human recombinant GAL to the co‐culture system promoted the proliferation, migration, and invasion of SACC cells significantly, but inhibited the apoptosis of SACC cells. Adding M871, a specific antagonist of GALR2, significantly blocked the above effects (p < 0.05) and inhibited the PNI of SACC cells in vitro and in vivo (p < 0.05).

**Conclusions:**

This study demonstrated that nerve‐derived GAL activated GALR2 expression, and promoted EMT in SACC cells, thereby enhancing the PNI process. Interruption of the GAL/GALR2 axis might be a novel strategy for anti‐PNI therapy for SACC.

## Graphical abstract

Nerve‐derived galanin (GAL) could activate GALR2 and promote epithelial‐to‐mesenchymal transition in salivary adenoid cystic carcinoma (SACC) cells, thereby enhancing the perineural invasion (PNI) process. Interruption of the GAL/GALR2 axis might be a novel strategy for anti‐PNI therapy for SACC.

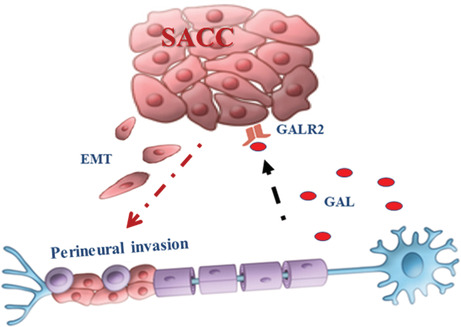

## 1 INTRODUCTION

Salivary adenoid cystic carcinoma (SACC) is a highly invasive malignancy arising from the salivary glands in the head and neck region.^1^ Perineural invasion (PNI) has been observed in up to 80% of SACC cases and correlates with nerve paralysis, pain, tumor recurrence, and distant metastasis.^2^, ^3^ In clinical scenarios, the negative surgical margins are frequently difficult to acquire due to nerve involvement, and thus local failure will occur. Unfortunately, there are no effective treatment strategies for PNI because the key mechanisms are largely unknown.^4^ Hence, it is warranted to identify key pathways highly correlated with PNI in SACC for optimal treatment selection and prognosis prediction.

Perineural invasion is a complex process mediated by multiple pathways^5^, ^6^ and has been shown to be associated with maladjusted genes^7^, ^8^ that regulate the release of tumor chemokines,^9^, ^10^ neurotropic factors,^11^ and adhesion molecules^12^ in nerve‐tumor microenvironment. Our preliminary research found the neurotrophic factors BDNF^13^ and NGF participated in the PNI process of SACC. However, blocking these factors just partially inhibited the invasion growth of SACC, which prompts us to dig neuropeptide to explore the full landscape of ‘nerve‐tumor crosstalk’ in SACC. Galanin (GAL) stimulates a variety of signal transduction and integration pathways by activating its receptors GALR1, GALR2, and GALR3,^14^, ^15^ and thus far, GALRs have been found to be the most effective markers for predicting the prognosis of head and neck squamous cell carcinoma (HNSCC) patients.^16^, ^17^ Previous studies have observed that a greater number of nerves were found adjacent to GALR2‐overexpressing murine tumors in HNSCC.^18^ However, the role of the GAL/GALR2 axis in SACC‐PNI has not been elucidated.

In addition, PNI is also associated with epithelial‐to‐mesenchymal transition (EMT).^13^, ^19^, ^20^ The transformation of epithelial cells into mesenchymal phenotypes through a specific procedure is known as EMT.^21^, ^22^ Through EMT, epithelial cells lost their cell polarity and acquired a high interstitial phenotype of migration and invasion, resistance to apoptosis, and degradation of the extracellular matrix.^23^, ^24^, ^25^ Although there have been studies on the relationship between nerve growth factor,^26^ neurotrophic factor,^27^ and EMT, there is no report on whether GAL regulates SACC through EMT. It may have potential exploration value to elucidate the mechanism between GAL and EMT in SACC‐PNI.

In the present study, we aim to clarify the mechanism of the GAL/GALR2 axis in SACC by a variety of in vivo and in vitro experimental models. The results showed that GAL and GALR2 were highly expressed in SACC‐PNI tissues, and their expression levels were significantly correlated with poor prognosis in SACC patients. Based on sequencing results, correlation analysis of differentially expressed genes demonstrated a close relationship between GAL and EMT molecules. In conclusion, our research found that the neurogenic GAL could induce EMT by activating its specific receptor GALR2 on SACC cells, and then promote the occurrence of PNI, which accelerates the malignant process of SACC. The above findings might provide a potential treatment strategy for SACC patients.

## 2 MATERIALS AND METHODS

### 2.1 Clinical data and samples

Patients in this study were selected from the study cohort of SACC patients in our center. Eligibility criteria included: age ≥18 years old; newly diagnosed histologically confirmed ACC; local–regionally advanced‐stage III/IV AJCC seventh edition; disease was determined resectable. Eastern Cooperative Oncology Group (ECOG) performance status ≤1; and adequate organ function. All patients received surgery and adjuvant radiotherapy. Survival status of patients was got from routine follow‐up and telephone follow‐up. SACC tissues were acquired at the Third Affiliated Hospital of the Fourth Military Medical University (FMMU) between 2010 and 2015, with the agreement of the FMMU's Medical Research Ethics Committee. Immunohistochemistry (IHC) was performed on 92 SACC samples, 50 normal salivary gland samples, and 20 normal nerve tissues that had been identified and paraffin‐embedded. Three cases of fresh tissues of SACC and normal salivary glands were obtained and stored in liquid nitrogen for use in transcriptome sequencing analysis. Informed consent was obtained from all SACC patients. The ethical approval number for animal experiments is IACUC‐20190627, and the ethical approval number for human samples is IRB‐REV‐2019051.

### 2.2 Immunohistochemistry

Four groups of tissue sections were selected for IHC staining in this experiment. Cut the paraffin‐embedded samples into 4 μm for use. Following the use of primary antibodies including GAL (Invitrogen; 1:50), GALR2 (GeneTex; 1:200), E‐cadherin (GeneTex; 1:500), vimentin (GeneTex; 1:500), and ki67 (GeneTex; 1:500) at 4°C overnight, the samples were stained with 3′‐diaminobenzidine and hematoxylin after being treated with peroxidase‐conjugated goat anti‐rabbit IgG as the secondary antibody. The staining index was calculated in five different fields at random (400× magnification). The final score was calculated by multiplying the staining intensity (weak = 1, intensive = 2) by the rate of positive staining (<25% = 1; 25%–50% = 2; 50%–75% = 3; >75% = 4). Finally, low expression (+) receives 1, 2, and 3 points. Scores 4, 6, and 8 for high expressiveness (++).

### 2.3 Sequencing and data filtering

Transcriptomics sequencing was conducted by Beijing Novogene Technology Co. Ltd. using the Agilent 2100 BioAnalyzer to detect RNA integrity and total amount accurately. Following library construction, preliminary quantification was performed using a Qubit2.0 Fluorometer, the insert size of the library was measured using an Agilent 2100 BioAnalyzer, and the effective concentration of the library was properly quantified using quantitative real‐time polymerase chain reaction (qRT‐PCR). Performed Illumina sequencing after the library passed inspection. DEseq2 (1.20.0) software was used to analyze differential expression between two study pairs (three biological replicates per group). Cluster Profiler (3.4.4) software was used to perform functional enrichment analysis for the Gene Ontology and pathway enrichment analysis for the Kyoto Encyclopedia of Genes and Genomes. qRT‐PCR was used to assess the dependability of the sequencing data.

### 2.4 Cell culture

Cultures were incubated in a humidified incubator at 37°C with 5% CO_2_. Human SACC‐83 and SACC‐LM cell lines were grown in RPMI‐1640 media supplemented with 100 U/ml penicillin, 100 mg/ml streptomycin, and 10% fetal bovine serum (FBS) (from Peking University School of Stomatology). Human SH‐SY5Y cell line was maintained in dulbecco's modification of eagle's  media with 100 U/ml penicillin, 100 mg/ml streptomycin, and 10% FBS.

### 2.5 Dorsal root ganglion culture

Dorsal root ganglia (DRG) were dissected from newborn BALB/c mice within 30 min after euthanasia and placed in 15 μl of Matrigel on a culture plate. Cultures were maintained with 5% CO_2_ at 37°C for 48 h to observe neurite growth.

### 2.6 Transwell co‐culture system

Seeded SACC were cells in the Transwell lower chamber, and SH‐SY5Y cells were seeded in the upper chamber of the 0.4 μm pore membrane (Corning). For the in vitro PNI co‐culture system, seeded DRG in the lower chamber, and SACC cells were planted in the upper chamber with a 0.4 μm pore membrane (Corning). The co‐culture system was maintained at 37°C with 5% CO_2_ in RPMI‐1640 media with 0.1% bovine serum albumin.

### 2.7 Enzyme linked immunosorbent assays

Use human galanin (GAL) ELISA Kit (CUSABIO, China) to assess the amount of GAL in the medium for each group according to the manufacturer's protocol.

### 2.8 qRT‐PCR assays

Using TaKaRa MiniBEST Universal RNA Extraction Kit (Takara Bio, Inc.) to extract total RNA from SACC tissues and SACC cells. To reverse RNA samples to cDNA, we used Prime Script RT Master Mix (Takara Bio, Inc.). qRT‐PCR was done with the SYBR Premix Ex Taq Kit (Takara Bio, Inc.). All primer sequences obtained from Sangon Biotech are listed in Table 1.

**TABLE 1:** The mRNA sequences

mRNAs	Primer sequences	
	Forward (5′‐3′)	Reverse (5′‐3′)
GAL	ACGAGGCTGGACCCTGAACAG	TTCTTGTCGCTGAATGACCTGTGG
BDNF	GACACTTTCGAACACGTGATAG	TACAAGTCTGCGTCCTTATTGT
NGF	GCAAGCGGTCATCATCCCATCC	TCTGTGGCGGTGGTCTTATCCC
VGF	CTTCTGCTGATCAACGGGTTAG	CTACCGGCTCTTTATGCTCAG
β‐Actin	TGACGTGGACATCCGCAAAG	CTGGAAGGTGGACAGCGAGG
GALR2	GCTCATCCTCTGCGTGTGGTTC	TTTGCGGAAGTGCTTGGAGACC
E‐cadherin	AGGCCAAGCAGCAGTACATT	ATTCACATCCAGCACATCCA
N‐cadherin	AGGTTTGCCAGTGTGACTCC	TGATGATGCAGAGCAGGATG
Vimentin	TGAATGACCGCTTCGCCAACTAC	CTCCCGCATCTCCTCCTCGTAG

### 2.9 siRNA constructs and transfection

The synthesized siRNA included two different sequences targeting human GAL (as listed in Table 2) and siRNA‐NC. The Lipofectamine 2000 transfection agent (Invitrogen) was used to transfect siRNAs into SACC cells. To determine transfection effectiveness, use Western blot and qRT‐PCR. The transfected SACC cells were then utilized to test their functionality.

**TABLE 2:** The siRNA sequences

siRNAs	Primer sequences	
	Sense (5′‐3′)	Antisense (5′‐3′)
siGAL‐NC	GUGAGCGUCUAUAUACCAUdTdT	AUGGUAUAUAGACGCUCACdTdT
siGAL‐1	GCGCACAAUCAUUGAGUUUCUTT	AGAAACUCAAUGAUUGUGCGCTT
siGAL‐2	CCUGAAGUCAAACCUUAAGAU	AUCUUAAGGUUUGACUUCAGGTT

### 2.10 Cell Counting Kit‐8 assay

Salivary adenoid cystic carcinoma cells (100 μl/well) were seeded at a density of 2 × 10^3^ cells per well in 96‐well plates. GAL (Tocris) was added to each well in different concentration gradients, as well as the plates were pre‐cultured in an environment at 37°C with 5% CO_2_ for 0, 12, 24, 36, and 48 h. Using the CCK‐8 kit (FEI YANG BIO) to evaluate cell proliferation according to the manufacturer's instructions.

### 2.11 Flow cytometry assay

To measure the apoptosis rate of SACC cells, fluorescein annexin V‐fluorescein isothiocyanate/propidium iodide double labeling (BD Biosciences) was utilized according to the manufacturer's instructions. The CytomicsTM FC 500 was used for flow cytometry (Beckman Coulter).

### 2.12 Western blot assays

Extracted total cell proteins from each sample. After transferring the protein to membranes (EMD Millipore) and blocking with 5% skim milk for 1 h. Primary antibodies to GAL (Invitrogen; 1:1000), GALR2 (GeneTex; 1:2000), E‐cadherin (GeneTex; 1:1000), N‐cadherin (GeneTex; 1:1000), vimentin (GeneTex; 1:10,000), β‐actin (GeneTex; 1:10,000), and GAPDH (GeneTex; 1:10,000) were incubated at 4°C overnight. The secondary antibodies were then incubated for 1 h with horseradish peroxidase‐conjugated secondary antibodies (Thermo Fisher Scientific; 1:5000). To identify the protein bands, chemiluminescence (ECL; Thermo Fisher Scientific) was employed, and images were acquired using the ChemiDoc TM XRS system and Image Lab TM software (Bio‐Rad Laboratories, Inc.).

### 2.13 Scratch wound healing assays

Salivary adenoid cystic carcinoma cells were seeded in the Transwell plate's lower chamber. Cells were scraped along a pre‐drawn path until they covered 90% of the plate.

After incubation in conditioned medium for 12 h, images were photographed under an inverted microscope (Olympus) to measure the moving distance of cells.

### 2.14 Migration and invasion assays

Seeded SACC cells onto the Transwell 8 μm inserts (Corning Costar). The SACC cells on the inserts were properly fixed with 4% paraformaldehyde and stained with 1% crystal violet after co‐culture for 12 h. To count the number of cells that went through the Transwell membrane, five separate visual fields were randomly picked under an inverted microscope.

### 2.15 In vitro model of PNI

Dorsal root ganglion was isolated and inoculated in the medium, according to the Refs. [28, 29]. After being treated with living Cell‐Tracker Green CMFDA (Yeasen Biotech Co., Ltd.), SACC cells were seeded on DRG medium. Photographs were taken under the microscope at 12 h under bright and green fluorescent fields. The Image‐Pro Plus 6.0 (Media Cybernetics) software was used to calculate the cell invasion potential and nerve invasion area.

### 2.16 In vivo xenograft tumor model

The Fourth Military Medical University Committee approved the use of animals for research. Luciferase‐labeled SACC cells were suspended in 0.2 ml of RPMI‐1640 with no FBS and injected (5 × 10^6^ per mouse) around the sciatic nerve of 5‐ to 8‐week‐old nude BALB/c mice (male; Beijing WeiTong LiHua Animal Co. Ltd.). One week after the injection, the mice in the antagonist group orally received M871 (Tocris), and the control group received the same dose of placebo methylcellulose (Sigma) orally every third day for 4 weeks (*n* = 5). After 28 days, tumors were harvested, separated carefully, weighed, and used for immunohistochemistry and PNI analysis.

### 2.17 Data analysis

For statistical analysis, GraphPad Prism 8 (GraphPad Software) and SPSS 22.0 (IBM) were used. The expression of GAL and GALR2 was assessed using Fisher's exact test. The correlation between GAL, GALR2, and their clinicopathologic parameters were measured by Spearman's rank correlation coefficient test. The Kaplan–Meier analysis was used to examine survival curves. *p* ≤ 0.05 was considered to be statistically significant for the Student's *t*‐test and the one‐way analysis of variance test. Every experiment was carried out in triplicate.

## 3 RESULTS

### 3.1 Increased GAL and GALR2 expression suggest a poor prognosis in SACC

**FIGURE 1:**
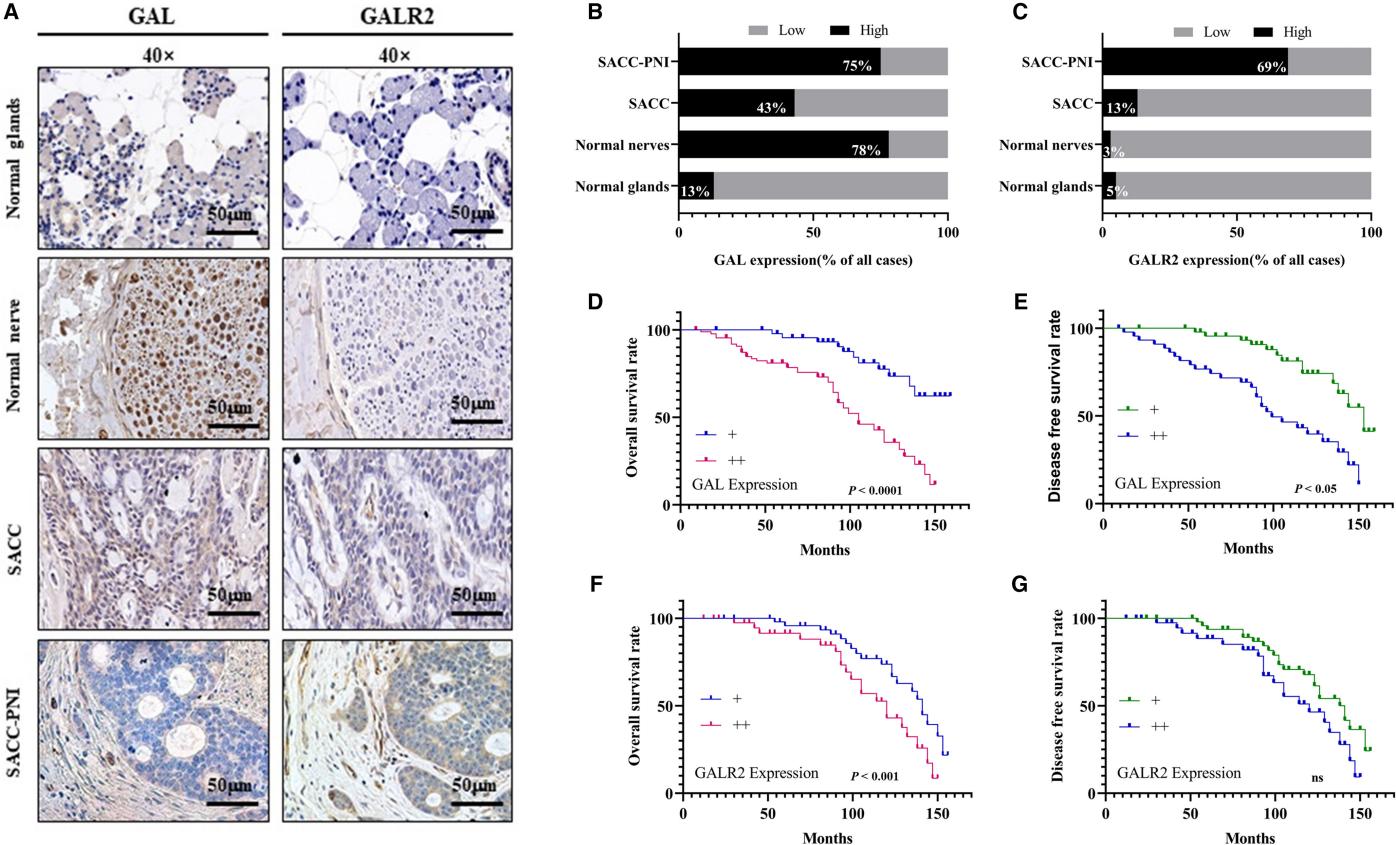
The expression of GAL and GALR2 in SACC and survival analysis. (A) IHC staining for GAL and GALR2 in clinical normal glands, normal nerves, SACC, and SACC‐PNI tissues (bar = 50 μm). (B, C) GAL and GALR2 expression levels in various SACC samples. (D, F) The overall survival (OS) rate of SACC is patients based on the expression of GAL and GALR2. (E, G) SACC patients' disease‐free survival (DFS) based on GAL and GALR2 expression. GAL, galanin; IHC, immunohistochemistry; ns, no significance; PNI, perineural invasion; SACC, salivary adenoid cystic carcinoma.

In this study, the expression of GAL and GALR2 in SACC was observed by IHC staining. GAL was expressed in SACC tissues, normal glands, and normal nerves. GALR2 was expressed in SACC tissues but was not detected in normal glands or normal nerves. The expressions of both GAL and GALR2 in SACC‐PNI tissues were significantly higher than in other groups (Figure 1A). The expression rates shown in Figure 1B,C exhibited the positive expression of GAL and GALR2 in different tissues intuitively. In addition, average optical density values of each group were calculated to assess the ratio of GAL and GALR2 in each group and reached the same conclusion as above.

The average follow‐up time for 92 SACC patients was 97.37 ± 38.54 months (mean ± standard deviation; range 9–162 months). At the end of this study, 41 patients (44.57%, 41/92) died, and 51 patients (55.43%, 51/92) were alive. Additionally, 31 patients (33.70%, 31/92) underwent distant metastasis. The difference in GAL and GALR2 expression was used to calculate the overall survival rate and disease‐free survival rate of SACC patients. Results showed that the high GAL and GALR2 expression were correlated with the poor prognosis of the SACC patients significantly (*p* < 0.05), although GALR2 expression was not associated with DFS (Figure 1D–G).

### 3.2 High expression of GAL is significantly associated with PNI in SACC patients

In SACC patients, GAL and GALR2 expression were significantly associated with stage, PNI, and distant metastasis (*p* < 0.05), but there was no statistical significance with Gender, Age, Site, or Histotype (as shown in Table 3).

**TABLE 3:** Association between GAL, GALR2 expression, and clinicopathological parameters of SACC patients

Variables	*n*	GAL expression		*r*_s_		GALR2 expression		*r*_s_	*p*‐value
		+	++		*p*‐value	+	++		
Gender									
Male	40	18	22	**−0.031**	**0.933**	21	19	**0.063**	**0.687**
Female	52	25	27			24	28		
Age									
>50	58	27	31	**−0.118**	**0.060**	30	28	**−0.098**	**0.052**
≤50	34	20	14			21	13		
Site									
Major	55	29	26	**0.040**	**0.291**	33	22	**0.112**	**0.103**
Minor	37	18	19			18	19		
Histotype									
S	31	14	17	**−0.038**	**0.019**	15	16	**0.024**	**0.016**
C/T	61	30	31			28	33		
Stage									
I + II	37	27	10	**0.359** **	**0.002** *	26	11	**0.245** *	**0.030** *
III + IV	55	20	35			25	30		
PNI									
−	57	36	21	**0.308** **	**0.003** *	37	20	**0.243** *	**0.006** *
+	35	11	24			14	21		
Metastasis									
−	61	37	24	**0.269** **	**0.001** *	39	22	**0.240** *	**0.001** *
+	31	10	21			12	19		

*Note*: Major: major salivary gland, Minor: minor salivary gland, S: solid type, C: cribriform type, T: tubular type. 1

Abbreviations: GAL, galanin; PNI, perineural invasion; SACC, salivary adenoid cystic carcinoma.

### 3.3 Neurogenic GAL activates GALR2 expression in SACC cells

**FIGURE 2:**
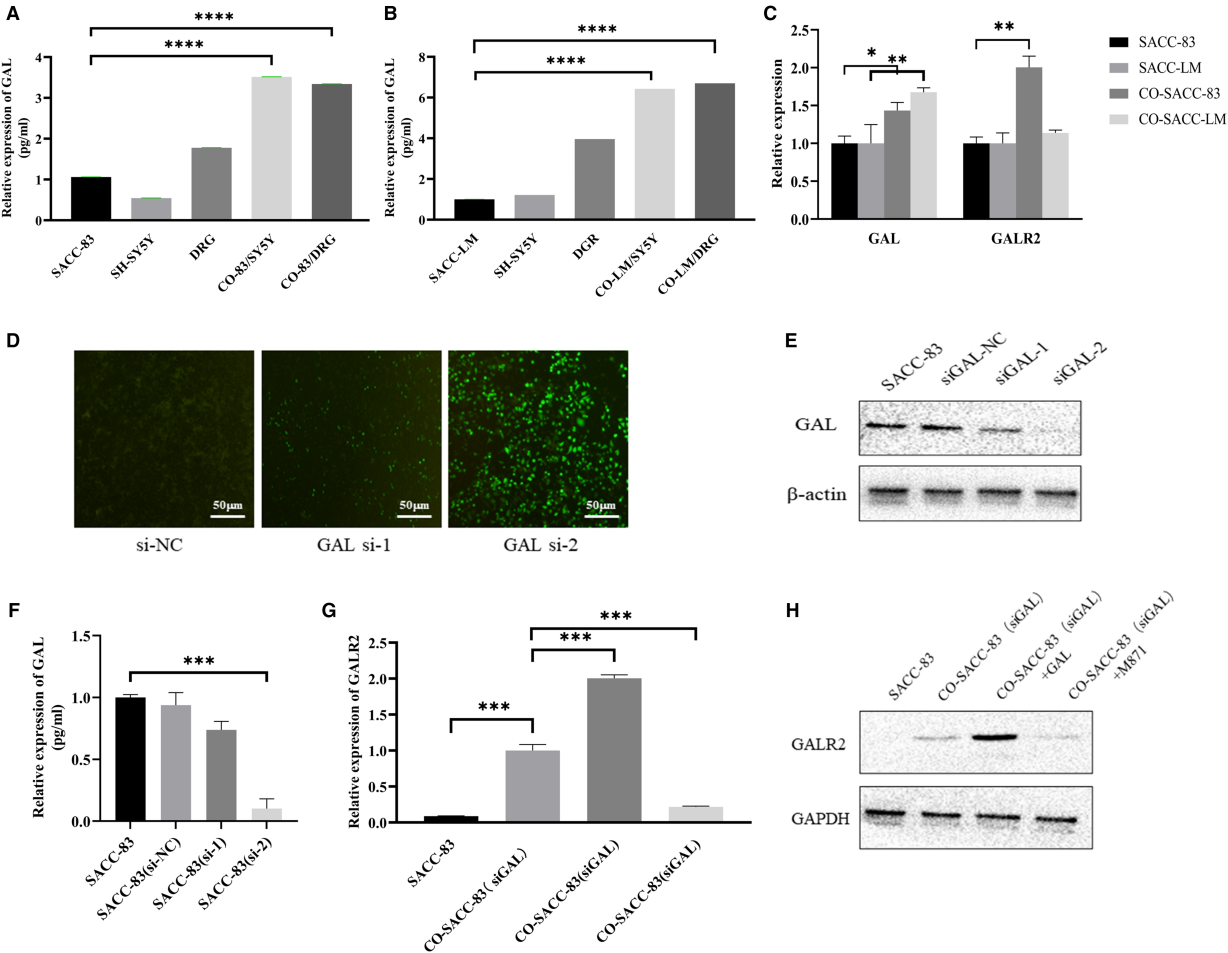
Neurogenic GAL could activate GALR2 in SACC cells. (A, B) ELISA results of GAL expression in SACC, SH‐SY5Y, and DRG. (C) qRT‐PCR results revealed GALR2 expression levels in SACC cells before and after co‐culture with SH‐SY5Y cells. (D–F) SACC‐83 cell transfection efficiency detection. (G, H) The expression of GALR2 was detected in various groups using Western blot and qRT‐PCR. DRG, dorsal root ganglion; ELISA, enzyme linked immunosorbent assay; GAL, galanin; qRT‐PCR, quantitative real‐time polymerase chain reaction; SACC, salivary adenoid cystic carcinoma. *p < 0.05, **p < 0.01, ***p < 0.001, ****p < 0.0001.

Our work further disclosed the molecular mechanism of GAL and GALR2 in SACC cells and nerve cells after confirming the expression of GAL and GALR2 in SACC and their clinical importance. First, SACC‐83 cells and SACC‐LM cells were co‐cultured with DRG tissue and SH‐SY5Y cells, respectively. The enzyme linked immunosorbent assay findings (Figure 2A,B) revealed that the concentrations of GAL and GALR2 in the co‐cultured group were significantly higher than in the control group (*p* < 0.05). Then, using Transwell co‐culture system to culture SACC cells and SH‐SY5Y cells. qRT‐PCR results (Figure 2C) revealed that GAL expression was significantly upregulated in co‐cultured SACC cells than in the singly cultured group (*p* < 0.05). GALR2 expression was markedly higher in co‐cultured SACC‐83 cells than in the singly cultured group (*p* < 0.05). In SACC‐LM cells, however, there was no significant change in GALR2 expression (*p* > 0.05).

In previous experiments, we found that GAL was expressed in both nerve tissues and SACC tissues. In order to exclude the influence of self‐expressed GAL in SACC cells in the co‐culture experiment, siRNA was used to downregulate the GAL expression (Figure 2D–F). The successfully transfected SACC‐83 (siGAL) cells were used for co‐culture experiments. The results showed that (Figure 2G,H) GALR2 expression was upregulated in the co‐culture group (*p* < 0.05) and the GAL cytokine group (*p* < 0.05). In contrast, GALR2 expression was significantly downregulated after the addition of the receptor inhibitor M871 (*p* < 0.05). These results suggest that neural‐derived GAL activated GALR2 in tumor cells and triggered the interaction between SACC and nerves.

### 3.4 The GAL/GALR2 axis promotes the invasion ability of SACC cells

**FIGURE 3:**
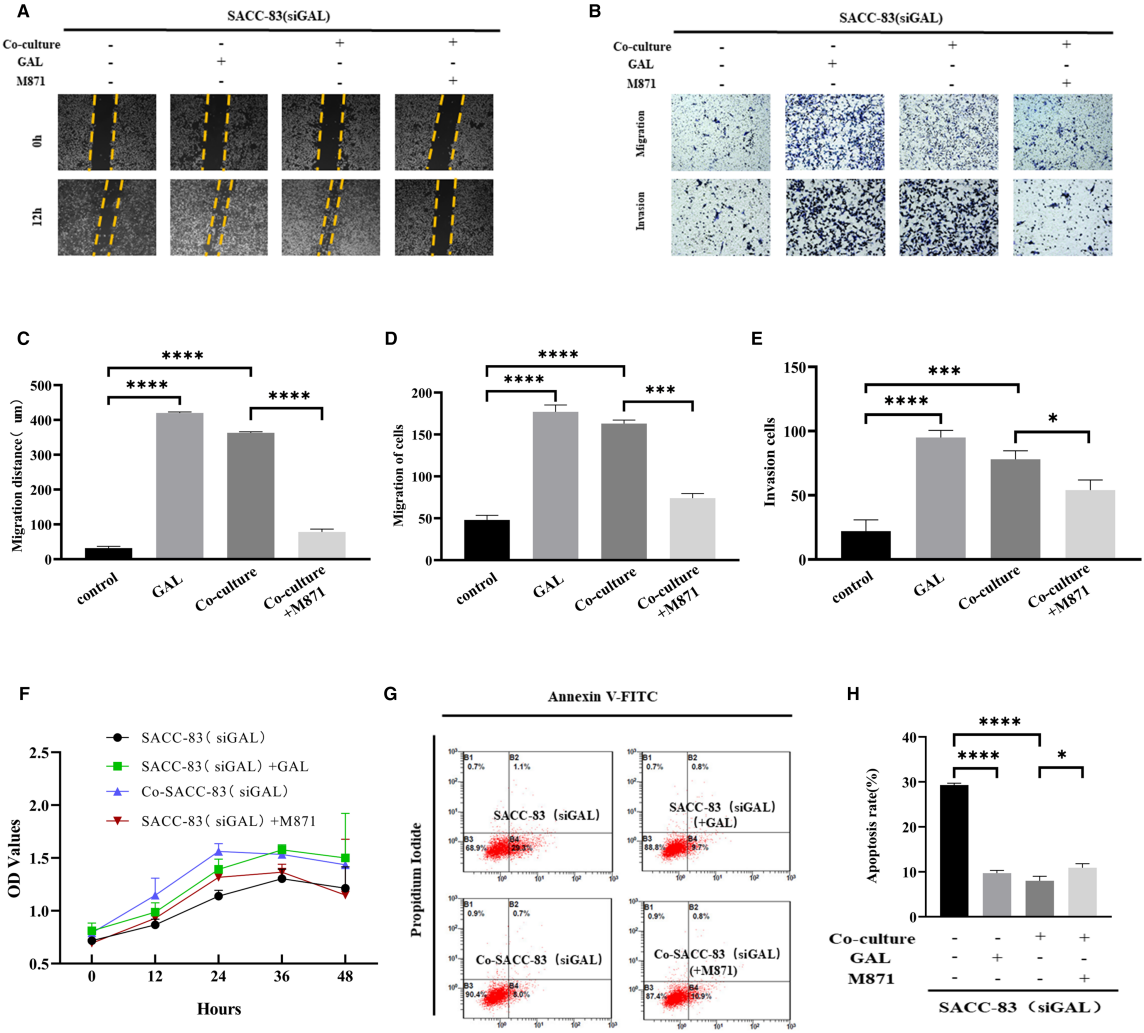
Stimulation of the GAL/GALR2 axis altered a variety of biological properties of SACC cells. The scratch wound healing assay (A, C), migration, invasion (B, D, E), proliferation (F), and apoptosis (G, H) experiments showed the effect of the GAL/GALR2 axis on the biological behavior of SACC cells under different treatment conditions. The magnification in the picture is 200×. GAL, galanin; SACC, salivary adenoid cystic carcinoma. *p < 0.05, ***p < 0.001, ****p < 0.0001.

The following experiments were carried out in this study in order to clarify which biological behaviors GAL/GALR2 is involved in SACC cells: Scratch wound healing assays (Figure 3A) and migration and invasion assays (Figure 3B) revealed that SACC cells' migration and invasion capacity were greatly boosted in co‐cultured groups. Additionally, using the GAL receptor inhibitor M871 could significantly impede the mobility of the SACC cells (*p* < 0.05) (Figure 3C–E).

We also performed proliferation and apoptosis experiments. It turned out that the effects of GAL/GALR2 on SACC cells were not statistically significant, but the apoptosis rate of SACC cells was affected by stimulating this axis (Figure 3F–H).

### 3.5 GAL promotes the EMT of SACC cells

**FIGURE 4:**
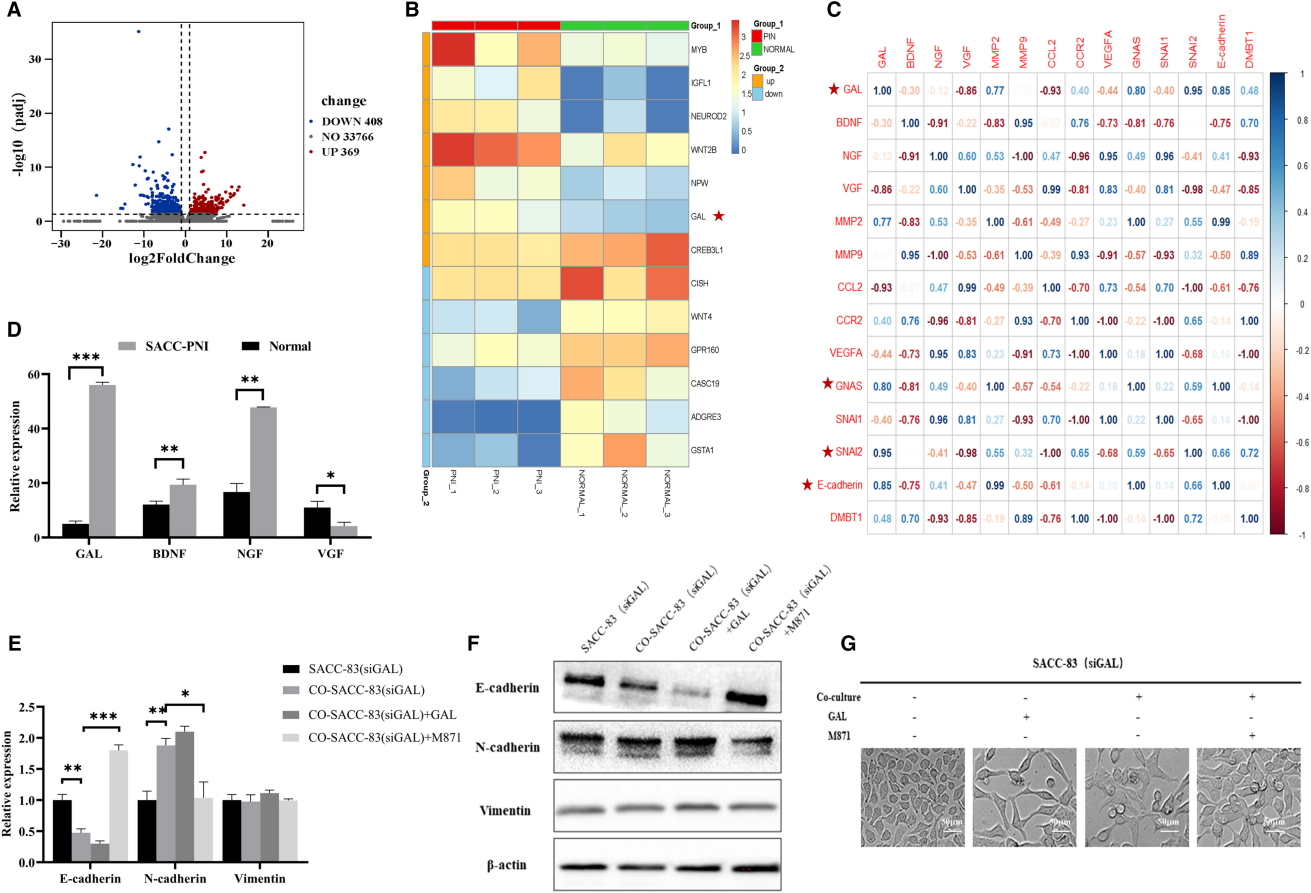
The GAL/GALR2 axis may play a crucial role in SACC via EMT. In transcriptome differential gene analysis, (A) Scatter plot of differential genes (*p*adj < 0.05, |log2FoldChange| > 0). (B) Heat map analysis of differential genes between two groups. (C) Heat map of gene correlation analysis. (D) Sequencing results were verified by qRT‐PCR. qRT‐PCR (E), Western blot (F), and cell imaging (G) results showed that the expression levels of EMT representative molecules were changed. The magnification in the picture is 400×. EMT, epithelial‐to‐mesenchymal transition; GAL, galanin; qRT‐PCR, quantitative real‐time polymerase chain reaction; SACC, salivary adenoid cystic carcinoma. *p < 0.05, **p < 0.01, ***p < 0.001.

Fresh SACC‐PNI tissues and normal salivary gland samples were collected for transcriptome sequencing in this study. We discovered 777 differentially expressed genes, 369 of which were upregulated and 408 were downregulated (Figure 4A). The expression of GAL was significantly upregulated compared to other molecules (Figure 4B). Then, we performed gene correlation analysis on GAL and other PNI‐associated molecules, such as neurotrophic factors (NT), matrix metalloproteinases, chemokines, and EMT expression markers. The result showed that GAL and its signal transduction pathway were highly correlated with EMT in SACC (Figure 4C). In the subsequent verification of sequencing results, we also found that compared with other NT, the differential expression of GAL was more significant (*p* < 0.05) (Figure 4D). In conclusion, GAL may play a crucial role in the progression of SACC and is strongly connected to EMT. The inner molecular mechanism between GAL and PNI in SACC needs to be studied urgently.

Whereafter, the co‐culture experiment found that regulating the GAL/GALR2 axis altered the expression of EMT‐related molecules, such as E‐cadherin, N‐cadherin, and vimentin. In particular, N‐cadherin expression was substantially higher in the co‐cultured group compared to the control group, but E‐cadherin expression was substantially lower in the co‐cultured group compared to the control group. Application of M871 significantly inhibited N‐cadherin expression in the co‐cultured group but promoted E‐cadherin expression in the solely cultured group (*p* < 0.05) (Figure 4E,F). Morphological observation showed that GAL promoted the spindle change of SACC cells, indicating the occurrence of EMT (Figure 4G). These findings imply that the GAL/GALR2 axis promotes epithelial‐to‐mesenchymal transition in SACC cells.

### 3.6 Blocking of GAL/GALR2 could inhibit the PNI of SACC

In order to observe the interaction between SACC cells and nerves more directly, this study also established an in vitro dynamic PNI model. We labeled SACC‐83 cells with CellTracker Green CMFDA that allowed SACC cells to be visualized under the green fluorescence field of the microscope. We can observe the interaction between SACC cells and DRG in co‐culture medium at 12 and 24 h, respectively. The results demonstrated that the application of exogenous GAL increased SACC‐83 cell invasion and migration to the DRG, as well as the growth and extension of neurites.

**FIGURE 5:**
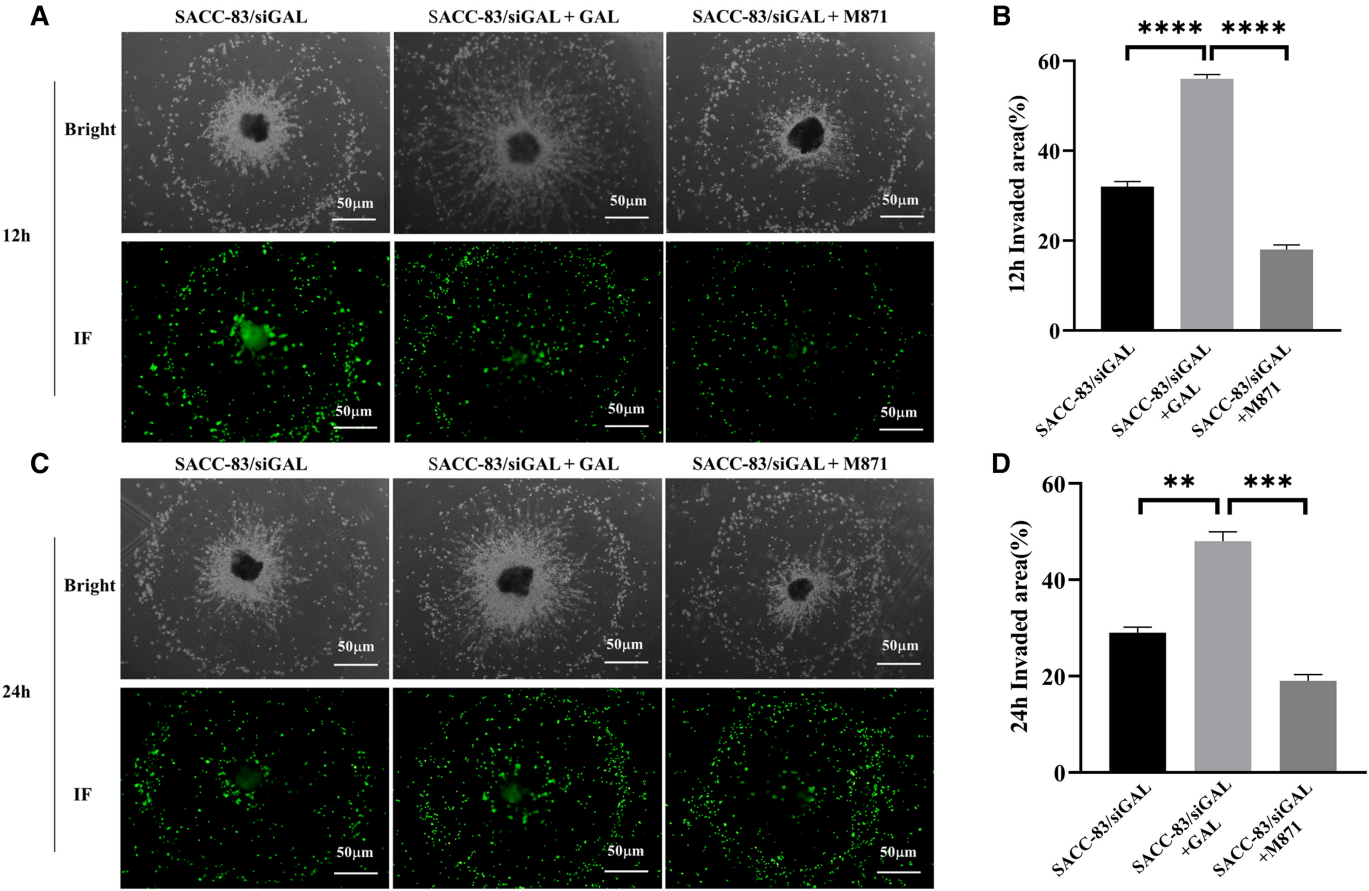
The establishment of an in vitro dynamic PNI model. Bright field and dark field observations were made after 12 h (A) and 24 h (C) of co‐culture. Statistical analysis (B, D) confirmed the experimental results. PNI, perineural invasion. **p < 0.01, ***p < 0.001, ****p < 0.0001.

M871 significantly inhibited the above phenomena (Figure 5). The in vitro results preliminarily indicate that the GAL/GALR2 axis involves multiple invasion and metastatic properties of SACC cells.

### 3.7 Application of GALR2 antagonist attenuates the tumorigenicity of SACC cells in vivo

**FIGURE 6:**
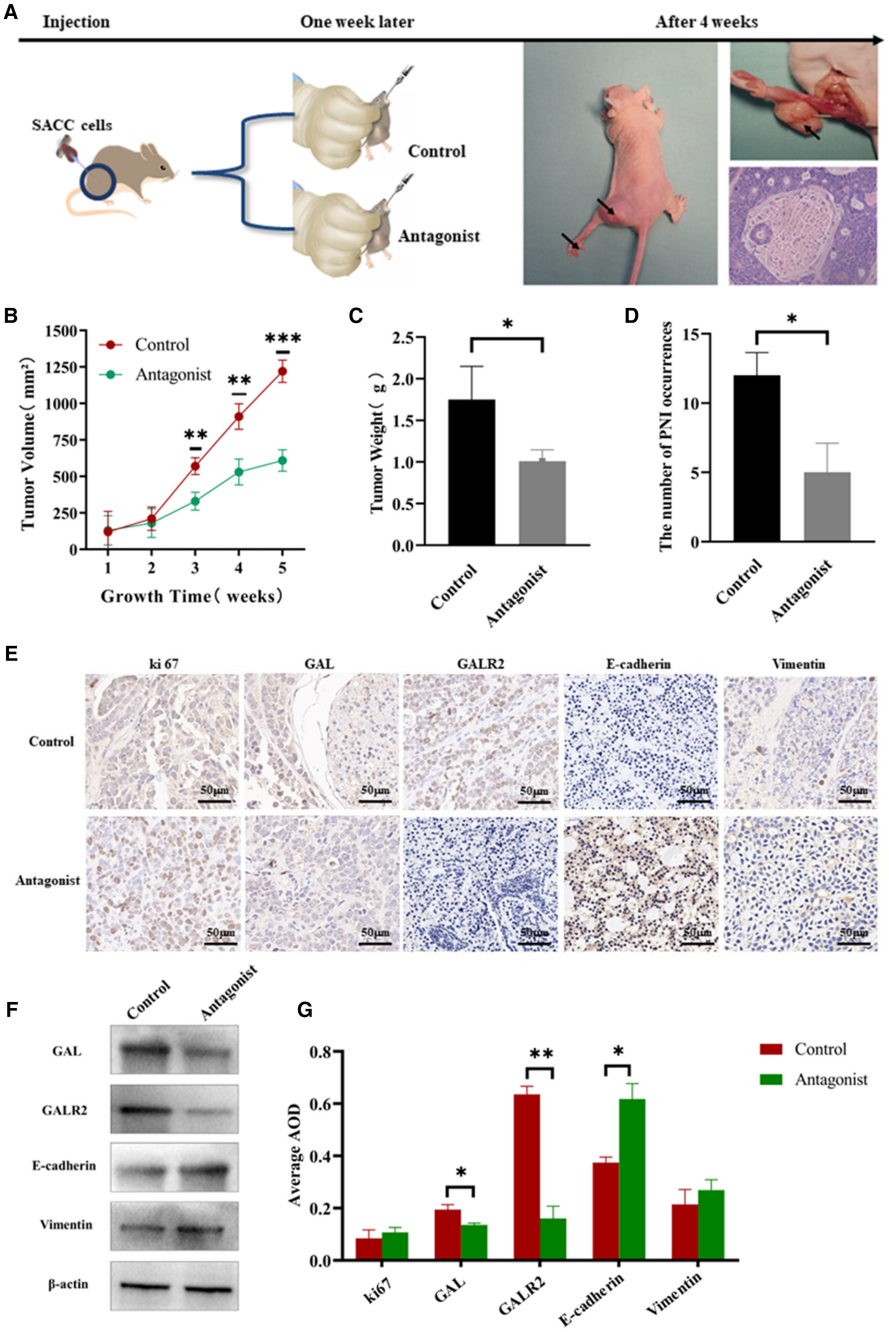
In vivo tumorigenesis experiment. (A) Establishment of the sciatic nerve PNI xenograft model and tumor specimen collection. (B) The tumor growth rate was monitored. (C) The tumor weight (D) and the incidence of PNI between the two groups were analyzed. (E) The expression rates of GAL, GALR2, and EMT‐related molecules were analyzed by IHC staining (*n* = 10, Positive = staining range ≥80%). (F) Immunohistochemical results were verified by Western blot. (G) Statistical analysis of immunohistochemical results. EMT, epithelial‐to‐mesenchymal transition; GAL, galanin; IHC, immunohistochemistry; PNI, perineural invasion. *p < 0.05, **p < 0.01, ***p < 0.001.

In this part, the control group and the antagonist group were designed to construct the SACC cell‐derived xenograft model. When the study was finished, the nude mice were euthanized, and the tumor samples were collected. Figure 6A shows the experimental procedure and tumor tissues obtained. PNI changes in the hind limbs of nude mice were also recorded and confirmed by hematoxylin and eosin staining. Compared with the control group, statistical results showed that M871 significantly reduced tumor growth (Figure 6B,C) and the incidence of PNI (Figure 6D) (*p* < 0.05). IHC staining was performed to verify the expression changes in GAL and GALR2, as well as the expression differences in EMT‐related molecules, which further confirmed the reliability of in vitro experiments (Figure 6E,G). In addition, we use Western blotting for verification above results (Figure 6F), the experimental data are in good agreement with the existing IHC results.

## 4 DISCUSSION

Perineural invasion is a prominent feature and a negative prognostic factor for several neurotrophic cancers, including pancreatic, prostate, SACC, and colon.^30^, ^31^ SACC is one of the most prevalent malignant tumors of the salivary glands. PNI leads to a poor prognosis for SACC patients. As the PNI mechanism is still unclear, there is still a lack of effective diagnostic and therapeutic approaches for PNI. Understanding the mechanism of PNI is therefore critical to increasing the survival rate of SACC patients. The current work discovered a previously unreported role of the GAL/GALR2 axis in the regulation of PNI in SACC.

Growing evidence demonstrates that nerves constitute an indispensable element in the cellular microenvironment of solid malignancies. Recent studies showed the nerves were not merely passive bystanders but significant players in the process of PNI. Nerves secrete a spectrum of neuroactive factors, including neuropeptides, neurotrophic factors, and neurotransmitters, to facilitate tumor progression in neurotrophic cancers such as pancreatic, prostate, and breast cancers. Previous studies have revealed that neurotrophic factors like BDNF and NGF contribute to the PNI process of SACC. However, blocking these factors just partially inhibited the tumor invasion, suggesting the need to dig into the secretion spectrum of nerves to reveal the full landscape of ‘nerve‐tumor synapse’ in SACC. GAL is widely distributed in the central and peripheral nerves, acting as a trophic factor for the central and peripheral nervous systems, and is highly involved in nerve injury and repair.^32^ In the context of tumors, GAL was found to promote tumor invasion via binding to its receptor, GALR2, in head and neck cancer.^18^ In the present study, we discovered that both GAL and GALR2 were overexpressed in SACC tissues and were substantially linked with SACC‐PNI. The in vitro data further showed that the neural‐derived GAL activated GALR2 to increase SACC cell proliferation, migration, and invasion while inhibiting apoptosis. Furthermore, both in vitro and in vivo, the GALR2 inhibitor M871 significantly blocked the PNI of SACC cells. The above findings demonstrate that GAL promotes the PNI process of SACC cells. Thus, our study reveals an unidentified role of GAL in the neural microenvironment of SACC and helps to better understand the mechanism of PNI in SACC.

Epithelial‐to‐mesenchymal transition is considered a typical sign of tumor invasion and metastasis.^33^ Numerous studies indicate that tumor cells undergo EMT in the process of PNI. A recent study found that nerve‐derived NGF induces EMT and confers tumor cell resistance to the EGFR inhibitor erlotinib in HNSCC.^34^ Our earlier research found that BDNF, a neurotrophic factor, induced EMT and enhanced SACC cell motility and invasion.^13^ Using transcriptome sequencing, we discovered that GAL expression was considerably raised in SACC tissues and that GAL expression patterns were closely linked with EMT pathways in SACC. In vitro nerve‐tumor co‐culture results showed that GAL increased the expression of N‐cadherin and Vimentin (mesenchymal biomarkers) while suppressing the expression of E‐cadherin (epithelial biomarker) in SACC cells. Besides, GAL promoted mesenchymal morphology and inhibited the epithelial morphology of SACC cells. Blocking of the GAL/GALR2 axis using the GALR2 inhibitor M871 significantly inhibited the above effects. Moreover, the in vivo study verified that M871 clearly decreased the expression of N‐cadherin and Vimentin while increasing E‐cadherin expression in tumor tissues. These findings indicate that nerve‐derived GAL conferred the EMT of SACC cells, thus stimulating the invasion of SACC.

Perineural invasion is acknowledged as the fourth route for tumor dissemination, apart from the three well‐known forms: straight invasion of surrounding tissue, lymphatic spread, and hematogenic spread.^35^ The knowledge of the mechanisms of PNI is still incomplete. Therefore, growing studies have focused on the mechanism of PNI to develop PNI‐specific diagnostic markers and therapeutic targets. The high expression of SEMA3D, an axon guidance molecule, was found to be correlated with the PNI of pancreatic ductal adenocarcinomas (PDA), and knockdown of SEMA3D significantly inhibited the metastasis of orthotopic PDA cells in mice.^36^ Other preclinical studies found that targeting NGF had a satisfactory anti‐tumor effect.^37^, ^38^ In this study, we found that both PNI and poor prognosis in SACC were associated with high expression of GAL and GALR2. M871 strongly suppressed the PNI of SACC cells in vitro and in vivo by blocking the GAL/GALR2 axis. Thus, GAL and GALR2 may be potential diagnostic biomarkers and therapeutic targets for SACC, which is refractory to current treatment methods, including radiotherapy, chemotherapy, and immunotherapy.

In conclusion, our findings demonstrated that the GAL/GALR2 axis promoted SACC‐PNI via modulating tumor cell EMT. Our study may provide a novel PNI‐specific diagnostic and therapeutic strategy for SACC via targeting the GAL/GAR2 axis.

## AUTHOR CONTRIBUTIONS

**Jun Wang:** Conceptualization (equal); data curation (equal); formal analysis (equal); investigation (equal); methodology (equal); software (equal); validation (equal); writing – original draft (lead); writing – review and editing (equal). **Zihui Yang:** Conceptualization (equal); formal analysis (equal); funding acquisition (equal); investigation (equal); methodology (equal); software (equal); supervision (equal); writing – review and editing (equal). **Yuanyang Liu:** Data curation (equal); formal analysis (equal); methodology (equal); validation (equal). **Huan Li:** Conceptualization (equal); methodology (equal); writing – review and editing (equal). **Xiangming Yang:** Formal analysis (equal); project administration (equal); software (equal). **Wanpeng Gao:** Validation (equal). **Qi Zhao:** Investigation (equal). **Xinjie Yang:** Conceptualization (equal); data curation (equal); formal analysis (equal); funding acquisition (equal); methodology (equal); project administration (equal); resources (equal); supervision (equal); writing – review and editing (equal). **Jianhua Wei:** Funding acquisition (equal); methodology (equal); resources (equal); supervision (equal).

## FUNDING INFORMATION

This work was supported by grants from National Natural Science Foundation of China for Prof. Xinjie Yang (grant nos. 82173165 and 82002867) and Prof. Jianhua Wei (grant no. 81973114). This work was also supported by National clinical research center for oral diseases (grant nos. LCB202008 and LCA202001).

## CONFLICT OF INTEREST

The authors have declared that no competing interest exists.

## ETHICS STATEMENT

The study was performed in accordance with the Declaration of Helsinki. The study has been approved by the Medical Research Ethics Committee of the Fourth Military Medical University.

## Data Availability

The datasets used and/or analyzed during the current study are available from the corresponding author on reasonable request.

## Footnotes

1 **p* < 0.05, ‐1 < r_s_ < 0 represents a negative correlation, 0 < r_s_ < 1 represents a positive correlation, ***p* < 0.01.
